# Comparative Analysis of the Nutrient Composition and Polyphenol Content of Organic and Sulphured Dried Apricots From Central Asia

**DOI:** 10.1155/ijfo/7273055

**Published:** 2026-08-18

**Authors:** Jamila Smanalieva, Janyl Iskakova, Nurzat Shaikieva, Anke Foerster, Anne Hellwig, Uwe Schwarzenbolz, Thomas Henle

**Affiliations:** ^1^ Department of Food Chemistry, Faculty of Chemistry and Food Chemistry, TUD Dresden University of Technology, Dresden, Germany; ^2^ Department of Food Science and Technology, Institute of Technology, Kyrgyz State Technical University After I. Razzakov, Bishkek, Kyrgyzstan, kstu.kg; ^3^ Environmental Engineering Department, Engineering Faculty, Kyrgyz-Turkish Manas University, Bishkek, Kyrgyzstan, manas.edu.kg; ^4^ Chemical Engineering Department, Engineering Faculty, Kyrgyz-Turkish Manas University, Bishkek, Kyrgyzstan, manas.edu.kg

**Keywords:** amino acids, antioxidant capacity, ascorbic acid, dried apricots, total polyphenols

## Abstract

Dried apricots are recognised as a nutritious snack and a significant agricultural commodity, supporting the livelihoods of small‐ and medium‐scale farmers in Central Asia. Despite economic and dietary importance, the nutritional composition of Central Asian dried apricots remains unexplored. This study evaluated the carbohydrate, protein, fat, amino acid, ascorbic acid, macro‐ and microelement contents, total polyphenol content and antioxidant capacity of dried apricots sourced from Kyrgyzstan, Uzbekistan and Türkiye. The analysed samples were rich in carbohydrates (31.6–57.9 g/100 g), with sorbitol, glucose, fructose and sucrose as the predominant sugars and sugar alcohols. Protein content was low, with aspartic acid and proline identified as the principal amino acids. The total polyphenol content ranged from 111.65 to 251.99 mg gallic acid equivalent (GAE)/100 g. Sulphur dioxide–pretreated samples have higher ascorbic acid and polyphenol contents, resulting in greater antioxidant capacity than unsulphured samples. Furthermore, the drying process reduced fruit moisture content, thereby increasing the concentration of minerals, such as calcium, potassium, magnesium, sodium and copper. Among the cultivars studied, Mursan Jalil, sourced from Kyrgyzstan, exhibited the highest sucrose content (48.25 g/100 g), the lowest sorbitol content and the highest phenolic content (212 mg GAE/100 g). These results advance the understanding of the composition of Central Asian dried apricots and underscore their potential health benefits, dietary value and relevance for food security and sustainable agricultural development.

## 1. Introduction

Apricots (*Prunus armeniaca* L.) are an economically important fruit crop, ranking sixth worldwide in production volume. Major producing countries include Türkiye, Iran, Uzbekistan, Italy, Spain and the United States (California), with global production reaching approximately 4.5 million tonnes in 2024 [1, 2]. Central Asia is particularly important as both the preliminary centre of origin and genetic diversity of apricot [3] and one of the world′s leading production regions [2]. In 2024, Uzbekistan exported approximately 526–540 thousand tonnes (kt) of apricots, followed by Tajikistan (313–348 kt) and Kyrgyzstan (57 kt), highlighting the region′s strong export potential [1]. Furthermore, all three countries ranked among the Top 10 exporters of dried apricots in 2023 and 2024 [2]. Approximately one‐third of global apricot production is processed into dried products. Consumption of dried fruits has increased steadily in recent years owing to growing consumer awareness of their nutritional and health benefits [4]. Dried apricots are an important dietary source of antioxidant phytochemicals, dietary fibre, vitamins and minerals [5]. Polyphenols are recognised as major antioxidant compounds [6] that enhance the nutritional quality of foods and can help chronic diseases [7, 8]. Macro‐ and micronutrient composition, polyphenol content and antioxidant capacity differ significantly among apricot cultivars, owing to genetic and phenotypic diversity [9].

In Central Asia, apricots are typically dried whole or halved directly in the sun on open trays, ground surfaces, or, in some traditional cultivars, on the tree itself, and farmers usually sell the dried fruit without preliminary commercial processing [2]. In Türkiye, by contrast, sun‐dried apricots commonly undergo additional commercial processing, including washing and controlled redrying, before retail packaging (see Section 2.1). Before sun‐drying, apricots are often treated with sulphur dioxide (SO_2_) or sulphite compounds to preserve their orange colour and accelerate the drying process; sulphuring is typically carried out by burning approximately 1.5–2 g of sulphur per kg of fresh fruit for 6–12 h [2]. By contrast, organic dried apricots are produced without SO_2_ treatment.

Food composition data for dried apricots are available in several major databases, including USDA Food Data Central, the Bundeslebensmittelschlüssel (BLS), EuroFIR and the Russian Food Composition Tables and Database (FCTB). According to the EuroFIR database, which integrates food composition data from 26 organisations across Europe, the United States and Canada, dried apricots contain (per 100 g): 14–44‐g moisture, 10.6–14.8‐g fructose, 0.2–16.1‐g glucose, 8.1–29‐g sucrose, 2.9–5.4‐g protein, 3–4.6‐g ash and 3–21.6‐g ‐total dietary fibre, providing 207–295 kcal per 100‐g edible portion [10, 11].

Despite Central Asia being both the primary centre of apricot diversity and a major supplier of dried apricots to international markets, compositional data for dried apricots from these regions are absent from these databases [12]. Beyond this lack of basic nutritional information, several important knowledge gaps remain. First, the effects of sulphuring and nonsulphuring on the nutritional composition of Central Asian dried apricots have not been systematically evaluated, despite sulphur dioxide treatment being a widely used post‐harvest practice that influences colour, antioxidant retention and shelf life. Second, cultivar‐specific nutritional characteristics remain largely unknown, although the exceptional genetic diversity of Central Asian apricots suggests substantial variation in sugar composition, crude fibre and polyphenol content. Third, compositional differences associated with geographical origin across Kyrgyzstan, Uzbekistan and Türkiye have not been comprehensively investigated, making it difficult to assess the influence of production region on nutritional quality. In addition, information on the amino acid (AA) composition of dried apricots from these regions is scarce.

The present study addresses these knowledge gaps through a comprehensive compositional analysis of dried apricots from Uzbekistan, Kyrgyzstan and Türkiye, including both sulphured and unsulphured products, multiple cultivars and different geographical origins. Samples from Türkiye were included as a reference producer, given that the country is the world′s largest exporter of dried apricots and represents an international benchmark for processing practices and quality. By analysing the same cultivars processed with and without sulphur dioxide, and by comparing multiple cultivars across three countries within a single analytical framework, this study provides the first systematic compositional dataset for Central Asian dried apricots and the first direct, within‐study comparison of how processing, cultivar and geographical origin independently and jointly shape their nutritional and phytochemical profile. This design moves beyond a purely descriptive survey, providing information that is directly relevant to breeding programmes, processors and food composition database curators seeking to predict or standardise the nutritional quality of dried apricot products.

## 2. Materials and Methods

### 2.1. Sample Preparation

Dried apricots (*P*. *armeniaca* L.) were obtained from farmers and local retailers in Kyrgyzstan and Uzbekistan. Samples of Turkish origin, including both sulphured and unsulphured sun‐dried apricots, were purchased from supermarkets in Germany. Three cultivars were included in the study: Suhany and Kurmaiy (both from Kyrgyzstan, the latter available as sulphured and unsulphured sun‐dried products), and Mursan Jalil, sourced from both Kyrgyzstan and Uzbekistan. Samples also differed in post‐harvest handling beyond sulphuring. Unsulphured status should not be interpreted as indicating organic production, as the two attributes were not necessarily associated in the samples analysed. The organic certification status of each sample is presented in Table 1; among all samples analysed, only the Suhany cultivar from Kyrgyzstan was certified as organic. Turkish samples had undergone commercial post‐harvest processing, including washing and controlled redrying to a residual moisture content of 20–25 g/100 g, before being packaged into consumer‐ready bags for retail sale. The vacuum‐packaged Suhany sample (SU‐vkg) additionally underwent washing and a short‐term microwave heat treatment prior to vacuum packaging.

**Table 1:** Samples′ cultivar, country of origin and abbreviations.

*N*	Cultivar	Country of origin	Processing method	Source (farm vs. retail)	Organic certificate	Sample abbreviation
1	Kurmaiy	Kyrgyzstan	Stone expressed, sun‐dried, unsulphured	Farm	No	Kus‐kg
2	Kurmaiy	Kyrgyzstan	Stone expressed, sun‐dried, sulphured	Farm	No	Ks‐kg
3	Suhany	Kyrgyzstan	With stone, sun‐dried, unsulphured	Fabrika Alysh‐Dan	Yes	SU‐kg
4	Suhany	Kyrgyzstan	Stone expressed, sun‐dried, unsulphured, softened, vacuum packaged	Fabrika Alysh‐Dan	Yes	SU‐vkg
5	Mursan Jalil	Kyrgyzstan	With stone, sun‐dried, unsulphured	Farm	No	MJ‐kg
6	Mursan Jalil	Uzbekistan	With stone, sun‐dried, unsulphured	Farm	No	MJ‐uz
7	Unknown	Türkiye	Stone expressed, sulphured, sun‐dried	Retail, packaged in Edeka, Germany	No	EDs‐tr
8	Unknown	Türkiye	Stone expressed, unsulphured, sun‐dried	Retail, packaged in KULTH, Germany	No	KUus‐tr

To minimise biological variability among individual fruits, a 500‐g composite sample was prepared for each cultivar by pooling multiple fruits collected from the same production source, a widely accepted approach for obtaining a representative estimate of average nutritional composition in food composition studies [13]. For the farm‐sourced samples (Kyrgyzstan and Uzbekistan), each composite sample originated from a single processing batch (lot size approximately 1–2 t of a given cultivar), assembled from fruit harvested from multiple trees within the same orchard. Although this represents a single production source rather than multiple independent farms, the inclusion of fruit from numerous trees within the batch captures a substantial degree of tree‐to‐tree biological variability, whereas the large batch size and composite sampling procedure minimise the influence of any single tree or fruit on the reported mean composition. The retail‐sourced Turkish samples, by contrast, represent pooled commercial lots of unknown orchard origin, processed and packaged as described above. Before analysis, the dried apricots were frozen in liquid nitrogen, manually ground into small fragments, transferred to sealed tubes and stored at −25°C until analysis. Sample abbreviations used throughout the study are listed in Table 1.

### 2.2. Determination of Moisture Content

The moisture content was determined by drying 3–5 g of each sample to a constant weight at 105^°^C ± 1^°^C using a halogen‐infrared moisture analyser (MBG 163L, VWR, Italy) according to the method described by [14].

### 2.3. Determination of Carbohydrates by High‐Performance Anion‐Exchange Chromatography With Pulsed Amperometric Detection (HPAEC‐PAD)

Glucose, fructose, sucrose and sorbitol contents were determined by HPAEC‐PAD (Dionex ICS‐6000 System, Thermo Fisher Scientific Inc., United States) following the method of Zhang et al. [15]. Approximately 10–13 mg of each sample was extracted with 10 mL of ultrapure water in an ultrasonic bath for 15 min. The extracts were subsequently filtered through Whatman No. 4 filter paper and diluted 20‐fold with ultrapure water before analysis.

Chromatographic separation was performed using two Dionex CarboPac PA1 analytical columns (2 × 250 mm) maintained at 20°C. Isocratic elution was carried out with a mobile phase consisting of 80% 200 mM NaOH and 20% ultrapure water at a flow rate of 0.25 mL/min. The injection volume was 10 *μ*L. External calibration curves were prepared for sorbitol, glucose, fructose and sucrose over the concentration range of 1–200 *μ*mol/L using analytical standards obtained from Carl Roth GmbH + Co. KG (Karlsruhe, Germany). Chromatographic data were processed using Chromeleon 7.2.10 software.

### 2.4. Determination of Protein Content

The protein content of dried apricot kernels and flesh was determined using the Kjeldahl method (AOAC 950.48 Method) with a Kjeldahl analytical system (Büchi Labortechnik AG, Flawil, Switzerland). A factor of 6.25 was applied to convert total nitrogen content into protein content [14].

### 2.5. Determination of AA Profile

AA concentrations were determined according to the method of Lautenschläger et al. [16] using an ion exchange chromatography AA analyser (S433 SYKAM Chromatographie Vertriebs GmbH, Fürstenfeldbruck, Germany) equipped with postcolumn ninhydrin derivatisation and an analytical cation exchange column (LCA K07/Li, 7 *μ*m, 4.6 × 150 mm; SYKAM Chromatographie Vertriebs GmbH, Fürstenfeldbruck, Germany). AAs were detected at wavelengths of 570 and 440 nm. Glutamic acid was quantified as the combined content of glutamic acid and glutamine, whereas aspartic acid was determined as the combined content of aspartic acid and asparagine. Tryptophan was not quantified because it is degraded during acid hydrolysis. Likewise, cystine and methionine were not determined owing to oxidative losses during sample preparation. Each sample was analysed in duplicate.

### 2.6. Determination of the Fat Content of Apricots

Crude fat content was determined using the Soxhlet extraction method according to AOAC Method 2003.05 [14]. Lipids were extracted with petroleum ether (Sigma‐Aldrich, Germany). Each sample was analysed in triplicate.

### 2.7. Determination of Fibre Content

The crude fibre content was determined according to AOAC Method 978.10. Briefly, the samples were subjected to sequential acid hydrolysis with 0.128 M H_2_SO_4_ followed by alkaline hydrolysis with 0.313 M NaOH. The resulting residue was dried to a constant weight and subsequently ashed [14]. Each sample was analysed in triplicate.

### 2.8. Determination of Ash Content

Ash content was determined according to AOAC Method 942.05 by incinerating the samples in a muffle furnace at 550°C for 6 h [14]. Each sample was analysed in triplicate.

### 2.9. Analysis of Mineral Elements

Mineral elements analysis was performed according to the method described in Smanalieva et al. [17]. Briefly, 0.15 g of the ashed sample was dissolved in 15 mL of 10% HNO_3_ and shaken for 30 s. The solution was filtered through No. 389 filter paper (Sartorius, Germany), and the filtrate was collected in a 50‐mL volumetric tube, diluted to volume with ultrapure water.

The concentrations of Na, calcium (Ca), potassium (K), magnesium (Mg), iron (Fe), copper (Cu), zinc (Zn) and manganese (Mn) were determined using inductively coupled plasma optical emission spectrometry (ICP‐OES) (Optima 7000 DV, PerkinElmer, Waltham, United States) operated at a radiofrequency power of 1400 W, with a plasma gas flow rate of 15.0 L min^−1^, an auxiliary gas flow rate of 0.2 L min^−1^ and a nebuliser gas flow rate of 0.65 L min^−1^. The following emission wavelengths (nm) were monitored using the axial plasma view: Fe (II), 238.204; Cu (I), 327.393; Zn (II), 206.200; and Mn (II), 257.610. The radial plasma view was used for Na (I), 589.592; Ca (II), 317.933; K (I), 766.490; and Mg (I), 285.213.

Calibration standards were prepared at concentrations of 0.5, 1.0 and 5 mg L^−1^ for Ca, Mg (Supelco, Merk, Germany); 10, 50 and 100 mg L^−1^ for K; and 0.01, 0.1 and 1 mg L^−1^ for Zn, Fe, Mn, Na and Cu (Merck Millipore, Germany). All measurements were performed in triplicate, and the reported values represent the mean signal intensities.

### 2.10. Analysis of Antioxidant Capacity

The antioxidant capacity of dried apricot extracts was evaluated using the DPPH (2,2‐diphenyl‐1‐picrylhydrazyl) free radical scavenging assay, as described by Hangun‐Balkir and McKenney [18]. Briefly, 1 g of dried apricot was ground under a nitrogen atmosphere to obtain particles with a diameter of ≤ 3 mm, extracted with 20 mL of 80% ethanol by stirring for approximately 20 min and centrifuged at 5000 rpm for 3 min. The resulting supernatant was used for antioxidant analysis. Serial dilutions of the extract (1–10 *μ*g·mL^−1^) were prepared in 80% ethanol. The mixtures were shaken and incubated at room temperature in the dark for 30 min. The control consisted of 2 mL of DPPH solution mixed with 2 mL of 80% ethanol. Absorbance was measured at 517 nm using a UV‐Vis spectrophotometer (Tecan Infinite 200 PRO, Tecan, Männedorf, Switzerland). The DPPH radical scavenging activity was calculated according to Equation (1).

(1) %Inhibition=Abscontrol–AbssampleAbscontrol·100,

where Abs_sample_ is the absorbance of the reaction mixture containing the sample extract after 30 min of incubation, and Abs_control_ is the absorbance of the control solution containing DPPH without the sample extract. Because the tested extract concentration range (1–10 *μ*g·mL^−1^) did not achieve inhibition above approximately 35%–45% for any sample, antioxidant capacity was expressed as the IC_30_ value, defined as the concentration of extract required to inhibit 30% of DPPH radicals, determined by linear regression analysis of inhibition percentage against extract concentration. All measurements were performed in triplicate.

### 2.11. Total Polyphenol Content (TPC)

The Folin–Ciocalteu method, according to Waterhouse, was used to determine the TPC [19]. Dried apricots were extracted with 10% (*v*/*v*) methanol at a sample‐to‐solvent ratio of 1:1 (*w*/*v*). The extract was homogenised and filtered through filter paper. An aliquot (1 mL) of the filtrate was mixed with 5 mL of Folin–Ciocalteu reagent and allowed to react for 5 min. Subsequently, 4 mL of 75 g L^−1^ sodium carbonate (Na_2_CO_3_) (Merck KGaA, Darmstadt, Germany) was added. The reaction mixture was incubated in the dark at room temperature for 30 min, and the absorbance was measured at 765 nm against a reagent blank using a UV‐Vis spectrophotometer (Tecan Infinite 200 PRO, Tecan, Männedorf, Switzerland). A calibration curve was prepared using gallic acid standard solutions (0.5–5 mg mL^−1^). The TPC was expressed as milligrams of gallic acid equivalents (mg GAE) per 100 g dry weight. All measurements were performed in triplicate.

### 2.12. Analysis of Ascorbic Acid Content

Samples were prepared according to a validated method developed by the State Investigation Institute of Saxony [20]. Briefly, 1.250 g of dried apricot sample was mixed with 15 mL of 1.5% metaphosphoric acid (Sigma‐Aldrich, Germany), shaken for 15 min and sonicated for 3 min. The extract volume was adjusted to 25 mL with 1.5% metaphosphoric acid and filtered through Whatman No. 4 filter paper, followed by a 0.45‐*μ*m regenerated cellulose (RC) membrane filter before HPLC analysis. Sugars were analysed using an Agilent 1100 HPLC system (Agilent Technologies, Santa Clara, California, United States) equipped with a diode‐array detector. Chromatographic separation was performed on a ProntoSIL Phenyl column (250 × 4.6 mm) maintained at 40°C. The mobile phase was prepared according to Smanalieva et al. [17]. The flow rate was 0.8 mL min^−1^, and detection was carried out at 243 nm. External calibration curves were prepared individually for each sugar using appropriate standard solutions over the validated concentration range. All samples were analysed in duplicate.

### 2.13. Statistical Analysis

Statistical analyses were performed using IBM SPSS Statistics for Windows, Version 20.0 (IBM Corp., Armonk, New York, United States). A two‐way analysis of variance (ANOVA) was conducted to evaluate the effects of cultivar and country on the nutritional composition of dried apricot samples. Mean comparisons were performed using Duncan′s multiple range test. Differences were considered statistically significant at *p* < 0.05. Principal component analysis (PCA) was performed separately for four blocks of variables: macronutrients, carbohydrates (sugars), AAs and minerals, in addition to an integrative analysis incorporating TPC and ascorbic acid. Data were standardised (*z*‐scores) prior to analysis, and PCA was performed using scikit‐learn (Python 3.12).

## 3. Results and Discussion

### 3.1. Macronutrients of Dried Apricots

Table 2 presents the macronutrient and carbohydrate content of dried apricots.

**Table 2:** Macronutrients of dried apricots (wet basis).

Samples	Moisture, g/100 g	Fat, g/100 g	Protein, g/100 g	Sorbitol, g/100 g	Glucose, g/100 g	Fructose, g/100 g	Sucrose, g/100 g	Crude fibre, g/100 g	Ash, g/100 g
Kus‐kg	8.91 ± 0.87Db	0.48 ± 0.03B	1.57 ± 0.35B	7.84 ± 1.34Bb	13.63 ± 0.80	7.57 ± 0.54	30.55 ± 0.52Ab	2.76 ± 0.12a	4.69 ± 2.10
Ks‐kg	7.33 ± 0.78Cb	1.34 ± 0.25E	3.1 ± 0.64C	5.38 ± 1.26Cb	12.22 ± 1.72	9.00 ± 1.69	23.44 ± 4.00Bb	2.82 ± 0.01a	4.69 ± 2.15
SU‐kg	10.05 ± 0.41Db	0.15 ± 0.01A	2.09 ± 0.79C	7.86 ± 0.47Bb	5.03 ± 3.01	3.29 ± 2.62	29.31 ± 4.01Ab	2.54 ± 0.26a	3.42 ± 0.36
SU‐vkg	14.29 ± 0.76Eb	0.19 ± 0.00A	1.81 ± 0.47C	4.39 ± 1.02Cb	13.42 ± 6.06	11.39 ± 5.52	33.11 ± 4.00Ab	2.54 ± 0.26a	4.74 ± 0.23
MJ‐kg	5.30 ± 0.13Bb	0.10 ± 0.01A	1.38 ± 0.02A	1.78 ± 0.50Db	2.61 ± 0.95	1.90 ± 1.28	39.18 ± 1.57Ab	2.70 ± 0.60a	3.87 ± 0.61
MJ‐uz	5.22 ± 0.16Aa	na	1.58 ± 0.30A	4.78 ± 2.76Cb	6.65 ± 0.25	2.29 ± 0.27	48.25 ± 0.14Aa	2.04 ± 0.14b	4.07 ± 0.19
EDs‐tr	22.13 ± 0.96Fc	0.54 ± 0.20C	2.01 ± 0.65B	15.18 ± 1.69Aa	12.86 ± 0.85	9.60 ± 0.91	9.12 ± 0.89Cb	2.25 ± 0.69b	3.00 ± 0.28
KUus‐tr	22.31 ± 0.74Fc	0.66 ± 0.01D	2.44 ± 0.78C	9.98 ± 0.49Ba	10.10 ± 0.33	6.89 ± 0.10	16.07 ± 0.05Cb	1.75 ± 0.12c	2.55 ± 0.25
Dried apricots sulphured [11]	30.9	0.51	3.39	na	33.1	12.5	7.89	7.3^a^	2.57

*Note:* Data are represented as means (*n* = 3) ± standard deviation (SD). For statistical analysis (comparison of data between the sample and countries), data are calculated on a dry‐weight basis (this is not shown). A–D—Mean values within the column (sample) with different letters are significantly different (*p* ≤ 0.05). a–c mean values within the column (countries) with different letters are significantly different (*p* ≤ 0.05).

Abbreviation: na, not analysed.

^a^Fibre, total dietary.

The moisture content of dried apricot samples varied significantly between countries (*p* ≤ 0.05). Turkish apricot samples exhibited higher moisture contents, resulting in a softer texture, consistent with the additional washing and redrying step applied to these samples (see Section 2.1). This additional processing step distinguishes the Turkish samples from the Kyrgyz and Uzbek samples, which were obtained directly from farmers or local retailers without comparable industrial post‐processing. Nevertheless, all samples complied with the quality standard for dried apricots (≤ 25.0 g/100 g moisture) [21]. Notably, both Mursan Jalil samples (MJ‐kg and MJ‐uz) exhibited markedly lower moisture contents (5.22–5.30 g/100 g) than the other sun‐dried samples (7–22 g/100 g). This is attributable to a distinctive characteristic of the Mursan Jalil cultivar, whose fruits dry naturally on the tree and are harvested already dried, without requiring further post‐harvest sun‐drying under the same conditions as the other cultivars. This natural drying on the branch likely accounts for the substantially lower residual moisture observed in these samples.

The protein content ranged from 1.38 to 3.11 g/100 g and was lower than the values reported for sulphured dried apricots in *FoodEXplorer* [10]. When expressed on a dry matter basis, protein concentrations ranged from 2.05 to 3.95 g/100 g and differed significantly among samples (*p* ≤ 0.05). According to Gurrieri et al. [22], fresh apricots contain approximately 1.5 g/100 g of nitrogenous compounds, of which about 75% represents the protein fraction.

Sulphur dioxide pretreatment significantly affected the fat content (*p* ≤ 0.05). The fat content of the apricot samples ranged from 0.10 to 1.34 g/100 g, with the highest value observed in the sulphured cultivar Kurmaiy (Ks‐kg). This value was markedly higher than in the remaining samples and may reflect cultivar‐specific lipid accumulation, seed/kernel fragment contamination during grinding, or a sulphuring‐related effect requiring further investigation. Excluding this outlier, fat content was low and consistent across samples (0.10–0.66 g/100 g), with a median of 0.48 g/100 g, closely matching the 0.51 g/100 g reported for sulphured dried apricots in most FCDB [10, 11]. Alajil et al. [23] reported lower fat contents ranging from 0.11 to 0.27 g/100 g in six fresh apricot genotypes from India and Europe, although this comparison should be interpreted with caution given that fresh fruit typically has lower fat content per 100 g than dried fruit owing to the concentrating effect of moisture removal.

The carbohydrate profile included sucrose, glucose, fructose and sorbitol (Table 2). Sorbitol concentrations ranged from 1.8 to 15.2 g/100 g, with the lowest level observed in cv. Mursan Jalil (MJ‐kg). Sorbitol concentrations expressed on a dry matter basis differed significantly among cultivars and between countries (*p* ≤ 0.05). Sorbitol is a sugar alcohol that contributes to the overall sensory quality (sweet taste) of apricots while providing a lower glycaemic response and energy value than sucrose [24–26]. Because sorbitol is only partially absorbed in the small intestine, excessive intake may cause gastrointestinal discomfort, particularly at doses exceeding approximately 20 g/day [27–29].However, information on sorbitol concentrations in fruits and berries remains limited in many food composition databases, highlighting the importance of generating reliable compositional data for different apricot cultivars.

Glucose and fructose contents of the dried apricot samples ranged from 2.6 to 13.6 g/100 g and from 1.9 to 11.4 g/100 g, respectively. When expressed on a dry matter basis, glucose and fructose concentrations differed significantly among cultivars and between countries (*p* ≤ 0.05). Sucrose was the predominant sugar with concentrations ranging from 9.12 to 48.25 g/100 g. The total content of mono‐ and disaccharides ranged from 31.6–57.9 g/100 g. Central Asian dried apricots generally contained higher total sugar contents than dried apricots reported from Türkiye and the United States [4]. Gurrieri et al. [22] reported total sugar contents of 4.8 to 16.7 g/100 g in fresh apricots, whereas Alajil et al. [23] found values ranging from 9.79 to 15.59 g/100 g. Similarly, Kafkaletou et al. [30] observed higher sucrose concentrations in Greek cultivars (7.99 g/100 g FW) than in French cultivars (5.72 g/100 g FW), suggesting that both genotype and growing conditions influence sugar accumulation. In addition to environmental factors, differences in ripening stage and cultivar characteristics may also contribute to the observed variation in sugar composition [31].

The crude fibre content of the dried apricot samples ranged from 2.5 to 2.8 g/100 g, which was lower than the dietary fibre value of 7.3 g reported in FoodData Central [11]. This difference is expected because crude fibre represents only a fraction of total dietary fibre, primarily the insoluble components, whereas dietary fibre also includes soluble polysaccharides. On a dry matter basis, crude fibre content differed significantly according to geographical origin (*p* ≤ 0.05). Ash content, an indicator of the total mineral content of foods, ranged from 2.55 to 4.74 g/100 g in the investigated apricot samples. Although these values were generally higher than the 2.57 g/100 g reported for sulphured dried apricots in FoodData Central, ash content expressed on a dry matter basis did not differ significantly among the samples. Fresh apricots typically contain 0.50–0.89 g/100 g ash, depending on the cultivar [32], indicating that the drying process concentrates minerals together with sugars and other nutrients.

### 3.2. AA Profile of Dried Apricots

The AA profile of the dried apricot samples comprised 17 AAs (Table 3). Proline (0.079–0.536 g/100 g) and aspartic acid (0.07–0.386 g/100 g) were the predominant AAs, followed by glutamic acid (0.063–0.130 g/100 g), whereas the sulphur‐containing AAs cysteine and methionine were detected only at trace levels. In the present study, aspartic acid represents the combined content of aspartic acid and asparagine because asparagine is quantitatively converted to aspartic acid during acid hydrolysis.

**Table 3:** Amino acid profile of dried apricots (g/100 g).

Amino acids	Kus‐kg	Ks‐kg	SU‐kg	SU‐vkg	MJ‐kg	MJ‐uz	EDs‐tr	KUus‐tr	Apricots, dried, sulphured [11]
Asp + Asn^b^	0.144 ± 0.003	0.367 ± 0.035	0.156 ± 0.001	0.280 ± 0.095	0.070 ± 0.003	0.195 ± 0.006	0.386 ± 0.083	0.196 ± 0.145	0.937
Thr^a^	0.038 ± 0.001	0.046 ± 0.003	0.036 ± 0.004	0.047 ± 0.003	0.033 ± 0.001	0.050 ± 0.001	0.042 ± 0.001	0.026 ± 0.013	0.073
Ser	0.044 ± 0.000b	0.053 ± 0.004a	0.041 ± 0.004b	0.053 ± 0.004a	0.037 ± 0.001b	0.061 ± 0.003a	0.047 ± 0.001b	0.02 ± 0.013c	0.087
Glu + Gln^b^	0.097 ± 0.003b	0.121 ± 0.008	0.097 ± 0.006	0.119 ± 0.014	0.076 ± 0.001	0.130 ± 0.002	0.089 ± 0.002	0.063 ± 0.029	0.188
Gly	0.045 ± 0.002	0.052 ± 0.003	0.037 ± 0.004	0.044 ± 0.004	0.031 ± 0.001	0.053 ± 0.002	0.041 ± 0.000	0.027 ± 0.014	0.07
Ala	0.061 ± 0.002a	0.073 ± 0.005a	0.075 ± 0.004a	0.080 ± 0.009a	0.041 ± 0.001b	0.073 ± 0.002a	0.055 ± 0.001b	0.034 ± 0.020b	0.11
Cys	0.002 ± 0.002	0.001 ± 0.001	0.001 ± 0.001	0.002 ± 0.001	0.002 ± 0.001	0.002 ± 0.002	ND	ND	0.019
Val^a^	0.049 ± 0.002	0.059 ± 0.006	0.042 ± 0.004	0.051 ± 0.005	0.038 ± 0.001	0.060 ± 0.002	0.044 ± 0.002	0.208 ± 0.166	0.078
Met^a^	0.013 ± 0.001a	0.012 ± 0.001a	0.002 ± 0.002b	ND	0.002 ± 0.002b	0.012 ± 0.001a	ND	ND	0.015
Ile^a^	0.036 ± 0.001	0.045 ± 0.005	0.033 ± 0.003	0.039 ± 0.004	0.029 ± 0.001	0.046 ± 0.001	0.038 ± 0.001	0.026 ± 0.012	0.063
Leu^a^	0.064 ± 0.003	0.076 ± 0.004	0.057 ± 0.005	0.071 ± 0.007	0.051 ± 0.001	0.080 ± 0.003	0.065 ± 0.000	0.043 ± 0.023	0.105
Tyr	0.018 ± 0.001b	0.023 ± 0.001a	0.016 ± 0.001b	0.018 ± 0.001b	0.016 ± 0.001b	0.027 ± 0.001a	0.034 ± 0.001a	0.025 ± 0.006a	0.039
Phe^a^	0.059 ± 0.005	0.064 ± 0.005	0.036 ± 0.002	0.047 ± 0.004	0.035 ± 0.000	0.057 ± 0.001	0.048 ± 0.000	0.035 ± 0.013	0.062
His^a^	0.020 ± 0.003	0.030 ± 0.003	0.023 ± 0.002	0.029 ± 0.003	0.019 ± 0.001	0.028 ± 0.001	0.023 ± 0.001	0.012 ± 0.007	0.047
Lys^a^	0.031 ± 0.001c	0.050 ± 0.003b	0.044 ± 0.003b	0.051 ± 0.006b	0.035 ± 0.001c	0.076 ± 0.002a	0.053 ± 0.001b	0.033 ± 0.014c	0.083
Arg	0.016 ± 0.016d	0.035 ± 0.002b	0.029 ± 0.003c	0.036 ± 0.003b	0.026 ± 0.001c	0.041 ± 0.001a	0.049 ± 0.002a	0.036 ± 0.010b	0.066
Pro	0.176 ± 0.148c	0.286 ± 0.026b	0.097 ± 0.004d	0.452 ± 0.148a	0.142 ± 0.004c	0.079 ± 0.001d	0.319 ± 0.012b	0.539 ± 0.037a	0.821
Total sum of AA	0.9133 ± 0.392b	1.393 ± 0.110a	0.821 ± 0.045c	1.417 ± 0.311a	0.684 ± 0.018	1.070 ± 0.020b	1.334 ± 0.078a	1.331 ± 0.189a	
Max	0.176	0.367	0.156	0.452	0.142	0.195	0.386	0.539	
	Pro	Asp	Asp	Pro	Pro	Asp	Asp	Pro	
Min	0.002	0.001	0.001	ND	0.002	0.002	ND	0.000	
	Cys	Cys	Cys	Met	Met	Cys	Met	Cys, Val	
SUM of EAA	0.290	0.358	0.252	0.305	0.225	0.387	0.306	0.382	
% of EAA	31.75	25.69	30.69	21.52	32.89	36.16	22.93	28.70	

*Note:* Data are represented as means (*n* = 3) ± standard deviation (SD). a–d Mean values within the column with different letters are significantly different (*p* ≤ 0.05).

Abbreviations: Ala, alanine; Arg, arginine; Cys, cysteine; EAA, essential amino acids; Glu, glutamic acid; Gly, glycine; His, histidine; Ile, isoleucine; Leu, leucine; Lys, lysine; Met, methionine; ND, not detected; Phe, phenylalanine; Pro, proline; Ser, serine; Thr, threonine; Trp, tryptophan; Tyr, tyrosine; Val, valine.

^a^Essential amino acids.

^b^Asp + Asn aspartic acid + asparagine.

These findings are generally consistent with previous studies. Chen et al. [33] reported that proline, leucine and aspartic acid were the predominant AAs in dried Xiaobai apricots, followed by glutamine, serine and arginine. Similarly, Hamzaoğlu et al. [34] identified aspartic acid (0.229–0.455 g/100 g) as the major AA in dried apricots, followed by glutamic acid (0.556–0.791 g/100 g). Versari et al. [35] demonstrated that asparagine accounted for up to 78% of the total AA content of apricot juice (0.820–1.570 g/L), whereas Voi et al. [36] reported that it represented approximately 85% of the total free AA fraction in apricot purees (mean 0.49 g/100 g). The predominance of aspartic acid observed in the present study is therefore expected because acid hydrolysis converts asparagine into aspartic acid before analysis.

Overall, the results confirm that proline and aspartic acid are the principal AAs in dried apricots, whereas the concentrations of the remaining AAs are comparatively low. Differences in AA composition among cultivars are likely associated with genotype, fruit maturity, geographical origin and post‐harvest processing, including sulphur dioxide treatment.

It should be noted that tryptophan and the sulphur‐containing AAs cysteine and methionine could not be accurately quantified using the applied acid hydrolysis procedure because they are susceptible to degradation during hydrolysis. More reliable determination of these AAs requires preoxidation before hydrolysis, followed by analysis of their stable oxidation products using an appropriate analytical method.

### 3.3. Ascorbic Acid Content of Dried Apricots

**Figure 1:**
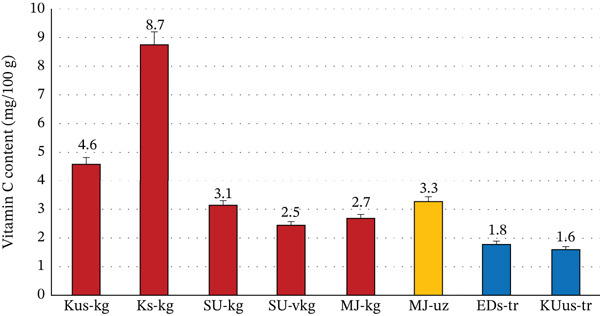
Ascorbic acid content of dried apricots and kernels: s, sulphured; us, unsulphited; v, vacuum‐packaged; red—Kyrgyzstan, blue—Türkiye and yellow—Uzbekistan.

The ascorbic acid content of dried apricots from three geographical regions is represented in Figure 1. The ascorbic acid content of sun‐dried apricot samples ranged from 1.6 to 8.7 mg/100 g, which is consistent with previously reported values for dried apricots [32, 37]. Similar concentrations (2.32–3.57 mg/100 g) were reported by Madrau et al. [38], whereas higher values (12.75–17.67 mg/100 g) were observed in dried apricot cultivars from Pakistan [39], indicating substantial variation among cultivars and growing conditions. Sulphur dioxide pretreatment markedly increased ascorbic acid retention in the Kurmaiy cultivar, with the sulphured sample (Ks‐kg, 8.7 mg/100 g) containing nearly twice the ascorbic acid content of its unsulphured counterpart (Kus‐kg, 4.6 mg/100 g), consistent with the protective effect of SO_2_ against oxidative degradation during drying. Notably, this effect was far less pronounced in the Turkish samples, where sulphured (EDs‐tr, 1.8 mg/100 g) and unsulphured (KUus‐tr, 1.6 mg/100 g) samples showed similarly low ascorbic acid contents. This is likely attributable to the additional commercial redrying step applied to the Turkish samples (see Section 2.1), during which further thermal degradation of ascorbic acid may occur regardless of sulphuring status, thereby masking the protective effect of SO₂ observed in the Kyrgyz samples. The two Mursan Jalil samples (MJ‐kg, 2.7 mg/100 g; MJ‐uz, 3.3 mg/100 g), which dry on the tree prior to harvest (see Section 2.1), showed intermediate and comparable ascorbic acid contents despite originating from different countries. This natural drying process occurs largely in the shade of the tree canopy with good air ventilation, in contrast to direct‐sun drying on open trays or ground surfaces used for the other cultivars, likely resulting in lower thermal and photo‐oxidative stress. This may explain the relatively consistent ascorbic acid retention observed in this cultivar regardless of geographical origin. Among the organic Suhany samples, the vacuum‐packaged sample (SU‐vkg, 2.5 mg/100 g) contained slightly less ascorbic acid than the no vacuum‐packaged sample (SU‐kg, 3.1 mg/100 g). This reduction is likely attributable to the additional washing and short‐term microwave heat treatment applied to the SU‐vkg sample prior to vacuum packaging, which may have caused partial thermal degradation of ascorbic acid despite the subsequent oxygen‐limited storage conditions. In addition to processing, environmental and biological factors such as cultivar, ripening stage, geographical region and altitude have been reported to influence ascorbic acid accumulation in apricot fruits [40, 41]. For example, Kan [41] found that apricots cultivated at higher altitudes (1490 m) contained higher concentrations of ascorbic acid than those grown at lower altitudes (825 m). Likewise, cultivated apricot varieties generally exhibit higher ascorbic acid content than wild species, in both fresh and dried fruits [37].

Overall, the present results confirm that the retention of ascorbic acid in dried apricots is determined by a combination of processing conditions and preharvest factors, with sulphiting providing the best preservation of the vitamin.

### 3.4. TPC of Dried Apricots

**Figure 2:**
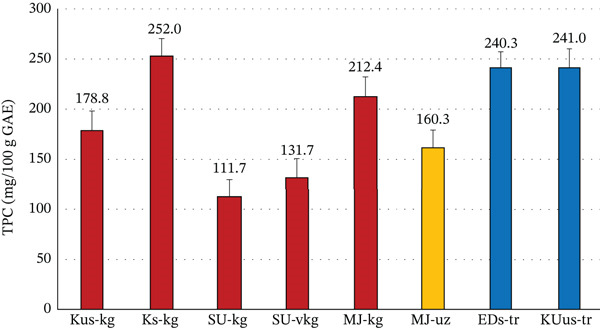
The total polyphenol concentration of dried apricots: s, sulphured; us, unsulphured; v, vacuum‐packaged; red—Kyrgyzstan, blue—Türkiye and yellow—Uzbekistan.

The TPC of the dried apricot samples ranged from 111.7 to 252.0 mg GAE/100 g (Figure 2). This is consistent with the range reported by Średnicka‐Tober et al. [42], who found mean TPC values of 231.04 and 283.99 mg/100 g for conventionally and organically produced dried apricots, respectively (drying method not specified). Significant differences in TPC were observed among cultivars, indicating that genotype was the primary factor affecting polyphenol accumulation. Sulphur dioxide treatment had a lesser effect on TPC. In particular, the sulphured cultivar Kurmaiy exhibited significantly higher TPC than the corresponding unsulphured samples, suggesting that sulphuring enhanced the retention of phenolic compounds by inhibiting polyphenol oxidase activity during drying. However, this protective effect was not evident in the Turkish cultivars.

The TPC values obtained in the present study were higher than those commonly reported for fresh apricots, reflecting the concentration effect associated with water removal during drying. Previous studies reported TPC values of 68.3–81.4 mg GAE/100 g fresh weight in wild apricots [43], 12.0–89.0 mg GAE/100 g in Hungarian cultivars [40], 25.31–89.95 mg GAE/100 g in Indian cultivars [23], approximately 48.5 mg GAE/100 g in Czech apricots [44] and 32.6–160.0 mg GAE/100 g in Spanish cultivars [45]. The observed variability among studies is likely attributable to differences in cultivar, environmental conditions, fruit maturity and analytical methodology [9].

It should be noted that the Folin–Ciocalteu assay estimates the total reducing capacity of the extract rather than the absolute concentration of phenolic compounds. Consequently, the measured TPC values may also reflect the presence of other reducing substances, such as ascorbic acid and reducing sugars.

### 3.5. Antioxidant Capacity

**Figure 3:**
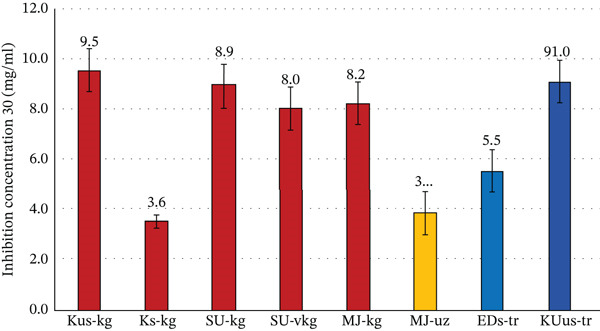
The antioxidant activity by DPPH (inhibitory concentrations IC_30_): s, sulphured; us, unsulphured; v, vacuum‐packaged; red—Kyrgyzstan, blue—Türkiye and yellow—Uzbekistan.

Dried apricots are an important source of antioxidants, including ascorbic acid, carotenoids, phenolic compounds and flavonoids, all of which contribute to their antioxidant properties [11, 46]. Among these compounds, phenolic acids and flavonoids are considered the major contributors to antioxidant activity because of their redox properties and ability to scavenge reactive oxygen species [47]. The antioxidant capacity determined by the DPPH assay (IC_30_) differed significantly between sulphured and unsulphured dried apricot samples (*p* < 0.05) (Figure 3). Sulphured apricots exhibited significantly greater antioxidant capacity than unsulphured samples, most likely because sulphur dioxide inhibited polyphenol oxidase activity during drying, thereby reducing enzymatic oxidation and preserving antioxidant compounds. It should be noted that residual sulphur dioxide can itself act as a reducing agent and may directly interfere with the DPPH assay, independently of its protective effect on endogenous antioxidant compounds; however, residual SO_2_ content was not quantified in this study. Total carotenoids, another class of antioxidants, were not analysed in this study and represent a target for future investigation.

Antioxidant capacity showed a moderate correlation with ascorbic acid content (*r* ^2^ = 0.579), whereas only a weak relationship was observed with TPC. A similarly weak association between antioxidant activity and TPC in apricots was reported by Sochor et al. [44], suggesting that antioxidant activity depends on the combined effects of multiple bioactive compounds rather than on TPC alone.

These findings indicate that dried apricots represent a valuable dietary source of natural antioxidants, particularly when appropriate drying and preservation methods are applied.

### 3.6. Macro‐ and Microelements of Dried Apricots

Table 4 presents the macro‐ and microelement composition of the dried apricot samples. The concentrations of K, Ca and Mg were generally consistent with previously published data.

**Table 4:** Mineral elements of dried apricots in mg/100 g.

Samples	Ca^a^	K^a^	Mg^a^	Na^a^	Cu	Mn	Fe	Zn
Kus‐kg	9.31cC	281.58d	10.04d	0.69dB	0.08c	0.03C	0.21dB	0.07
Ks‐kg	12.74cC	318.44d	13.05d	1.09dB	0.15c	0.04C	0.50dB	0.12
SU‐kg	43.78aC	836.69c	39.05b	2.09cB	0.48b	0.19C	1.87bB	0.42
SU‐vkg	39.40aC	556.23c	28.83c	1.72cB	0.27c	0.05C	0.55dB	0.34
MJ‐kg	44.33aC	1460.43b	42.74c	3.20bB	0.56a	0.25C	1.14cB	0.46
MJ‐uz	157.17aA	1805.02a	68.89a	6.12aA	0.50b	0.46A	3.60aA	0.47
EDs‐tr	61.19bB	1070.60b	47.10b	8.27aA	0.62a	0.28B	1.06cB	0.45
KUus‐tr	54.77bB	693.79c	31.04c	4.99bA	0.69a	0.18B	1.23c	0.46
Apricot, dried, sulphured [11]	55	1162	32	10	0.343	nd	2.66	0.39
Apricot, dried [48]	82	1370	50	11	0.800	1.5	4.4	0.400

*Note:* a–d mean values within the column with different letters are significantly different (*p* ≤ 0.05).

Abbreviation: nd, not detected.

^a^Macroelements.

Drying concentrates the minerals naturally present in fresh apricots by removing water. For example, fresh apricots contain approximately 231 mg K/100 g, whereas dried apricots typically contain up to 1162 mg/100 g [49]. In the present study, K concentrations ranged from 280 to 1805 mg/100 g, exceeding the values reported for dried apricots from Pakistan (490–520 mg/100 g) [39]. Particularly high K concentrations were observed in the Uzbekistani samples, which may be associated with differences in soil characteristics, agricultural practices, or fertiliser application. Considering the recommended daily K intake of 3500 mg [50], 100 g of dried apricots may provide approximately 8%–51% of the daily requirement.

Ca concentrations ranged from 9.3 to 157.17 mg/100 g, with the highest value observed in the Uzbekistani sample (MJ‐uz), consistent with the pattern already noted for K and Mg in this sample. Considering the recommended daily Ca intake of 1000 mg [50], 100 g of dried apricots may provide approximately 1%–16% of the daily requirement, depending on the cultivar and geographical origin.

Mg concentrations ranged from 10.04 to 68.89 mg/100 g, showing greater variability than previously reported for dried apricots from Australia, Türkiye and South Africa [39, 51]. Considering the recommended daily Mg intake of 350 mg [50], 100 g of dried apricots may provide approximately 3%–20% of the daily requirement, depending on the cultivar. The highest Mg concentrations were observed in the Uzbekistani sample (MJ‐uz), consistent with the pattern already noted for K and Ca, suggesting a shared soil‐ or fertiliser‐related origin for the elevated mineral content of this sample [52].

Sodium concentrations were relatively low (0.69–8.27 mg/100 g), remaining below the values reported for dried apricots from Australia and Pakistan [39, 51]. Consequently, the high K/Na ratio observed in the investigated samples represents a favourable nutritional characteristic associated with cardiovascular health [53].

The concentrations of essential trace elements, including Fe, Cu, Mn and Zn, were generally within the ranges reported in previous studies [49, 54]. Cu concentrations in cultivars KUus‐tr, EDs‐tr, SU‐kg, MJ‐kg and MJ‐uz exceeded those reported in FoodData Central [11] but remained lower than the values reported by Souci et al. [48]. Variations in trace element concentrations among samples are likely related to differences in cultivar, geographical origin, soil composition and cultivation practices.

Overall, the results demonstrate that drying substantially increases the concentration of both macro‐ and microelements in apricots. The observed differences among cultivars and countries of origin highlight the importance of genotype and growing conditions in determining mineral composition, confirming that dried apricots are a valuable dietary source of essential minerals, particularly K, Ca, Mg and Fe.

### 3.7. Multivariate Analysis

To complement the univariate comparisons presented in Sections 3.1–3.6 and to explore the overall structure of variation among samples, PCA was performed. Because residual moisture content varied substantially among samples (5.1–22.3 g/100 g), all analyses were performed on dry‐matter‐normalised data, allowing a moisture‐independent comparison of cultivar‐, sulphuring‐ and origin‐related effects:

#### 3.7.1. Macronutrients

**Figure 4:**
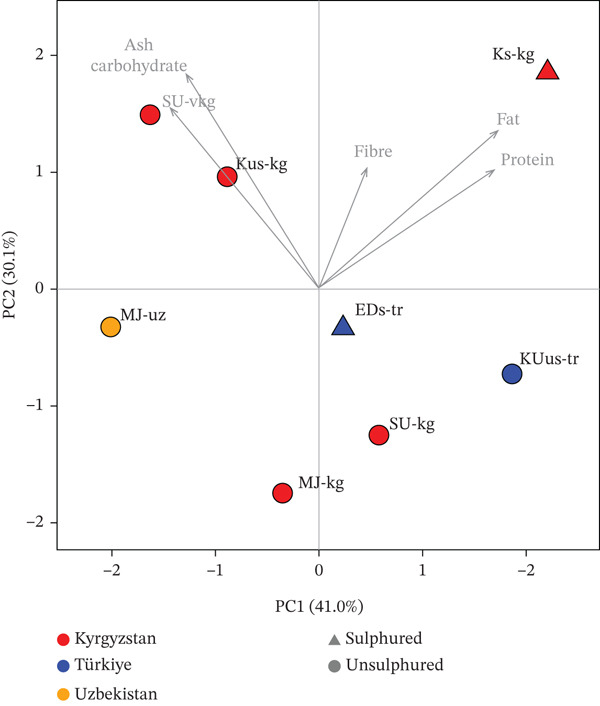
PCA—macronutrients: fibre, fat, ash, protein and carbohydrate content (dry matter basis).

PCA of five macronutrient‐related variables (fibre, fat, ash, protein and total carbohydrate content, calculated as the sum of sorbitol, glucose, fructose and sucrose; sample means, *n* = 8, dry matter basis) yielded two components explaining 41.0% and 30.1% of the total variance (71.1% cumulative) (Figure 4). PC1 contrasted fat and protein content against carbohydrate and ash content, with the sulphured Kurmaiy sample (Ks‐kg) and the unsulphured Turkish sample (KUus‐tr) occupying the positive extreme, and the Uzbekistani Mursan Jalil sample (MJ‐uz) occupying the negative extreme. PC2 was primarily associated with ash and carbohydrate content, separating the vacuum‐packaged Suhany sample (SU‐vkg) and the sulphured Kurmaiy sample (Ks‐kg), both comparatively rich in ash, from the Kyrgyz Mursan Jalil sample (MJ‐kg), which combined low fat and protein content with the lowest carbohydrate content among all samples. Sulphuring status did not consistently separate samples along either axis, supporting the conclusion that macronutrient composition, including total carbohydrate content, is primarily determined by cultivar rather than by sulphur dioxide treatment.

#### 3.7.2. Carbohydrates

**Figure 5:**
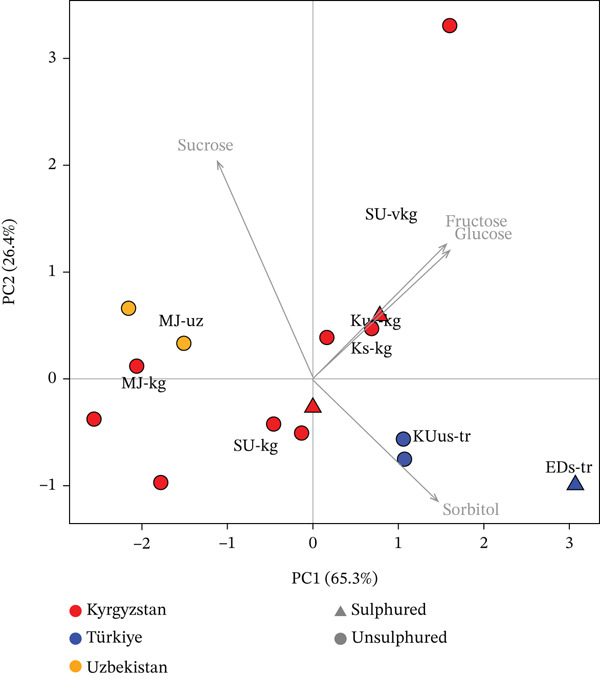
PCA—carbohydrates: sorbitol, glucose, fructose and sucrose (dry matter basis).

PCA was performed on the four measured sugars (sorbitol, glucose, fructose and sucrose) on a dry‐matter basis (Figure 5). The first two components explained 91.7% of the total variance, the highest proportion of explained variance among the individual variable blocks. PC1 (65.3%) contrasted glucose and fructose content against sucrose content, with the Kyrgyz Mursan Jalil sample (MJ‐kg) and the sulphured Turkish sample (EDs‐tr) occupying opposite extremes of this axis, reflecting their markedly different sucrose‐to‐reducing‐sugar ratios. PC2 (26.4%) was primarily driven by sucrose content, further distinguishing the vacuum‐packaged Suhany sample (SU‐vkg) from the remaining cultivars. As with macronutrients, sulphuring status did not produce a clear separation along either component, corroborating the univariate finding that sugar composition is primarily determined by cultivar rather than by sulphur dioxide treatment.

**Figure 6:**
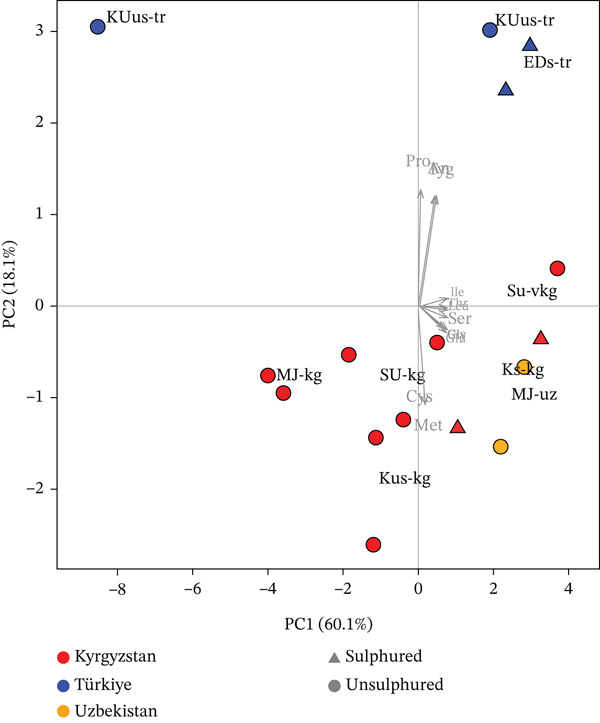
PCA—amino acid profile of dried apricots (dry matter basis).

To explore the overall structure of variation in AA composition among the dried apricot samples, PCA was performed on the standardised concentrations of the 17 detected AAs (Figure 6). The first two principal components accounted for 58.5% and 21.4% of the total variance, respectively, together explaining 79.9% of the variability in the dataset. PC1 was associated with the majority of AAs (including leucine, isoleucine, serine, threonine, glycine, glutamic acid, histidine, alanine, phenylalanine and lysine), which loaded positively and with comparable magnitude, indicating that this component largely reflects overall AA richness. Proline and valine loaded in the opposite direction along PC1, separating samples with a comparatively higher proportion of these two AAs from those dominated by the remaining AA fraction. PC2 was primarily defined by a contrast between arginine, tyrosine, aspartic acid and proline on one hand, and cysteine and methionine on the other, reflecting a secondary axis of variation independent of overall AA abundance.

Samples did not cluster clearly according to country of origin along either component, suggesting that, in contrast to the mineral profile (see Section 3.7), variation in AA composition is driven primarily by cultivar and processing factors rather than by geographical origin. The Uzbekistani sample (MJ‐uz) and the sulphured Kurmaiy sample (Ks‐kg) occupied the positive extreme of PC1, consistent with their comparatively high aspartic acid and glutamic acid contents (Table 3), whereas the unsulphured Turkish sample (KUus‐tr) occupied the negative extreme, largely as a consequence of its anomalously high valine content relative to the remaining samples. Given that this single value showed a disproportionately large standard deviation in the underlying dataset (Table 3), its influence on the ordination of KUus‐tr along PC1 should be interpreted with caution, and independent verification of this measurement is recommended before the pattern is generalised.

#### 3.7.3. Polyphenols, Ascorbic Acid and Antioxidant Capacity

**Figure 7:**
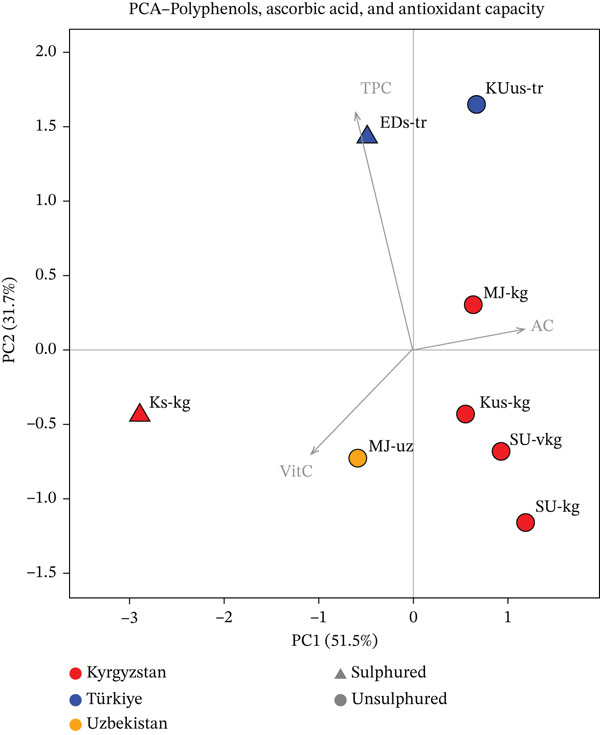
PCA—TPC, ascorbic acid and antioxidant capacity (IC_30_) (dry matter basis).

PCA of TPC, ascorbic acid content (both dry‐matter‐normalised), and antioxidant capacity (IC_30_, expressed per extract volume and therefore not moisture‐normalised) yielded two components explaining 51.5% and 31.7% of the total variance (83.2% cumulative) (Figure 7). PC1 contrasted antioxidant capacity (IC_30_) against ascorbic acid and TPC; because a lower IC_30_ value indicates stronger radical‐scavenging activity, this axis effectively separated samples with concurrently high polyphenol and ascorbic acid content and strong antioxidant activity from samples in which all three properties were comparatively low. The sulphured Kurmaiy sample (Ks‐kg) occupied the negative extreme of PC1, reflecting the simultaneous enhancement of TPC, ascorbic acid content and antioxidant capacity associated with sulphuring in this cultivar, consistent with the univariate findings reported in Sections 3.4 and 3.5. PC2 was primarily driven by TPC, distinguishing the Turkish samples (EDs‐tr and KUus‐tr), which combined comparatively high polyphenol content with moderate ascorbic acid content, from the remaining cultivars. Notably, the Uzbekistani Mursan Jalil sample (MJ‐uz) combined strong antioxidant activity with comparatively low TPC, indicating that high antioxidant capacity does not necessarily coincide with high TPC across cultivars.

#### 3.7.4. Minerals

**Figure 8:**
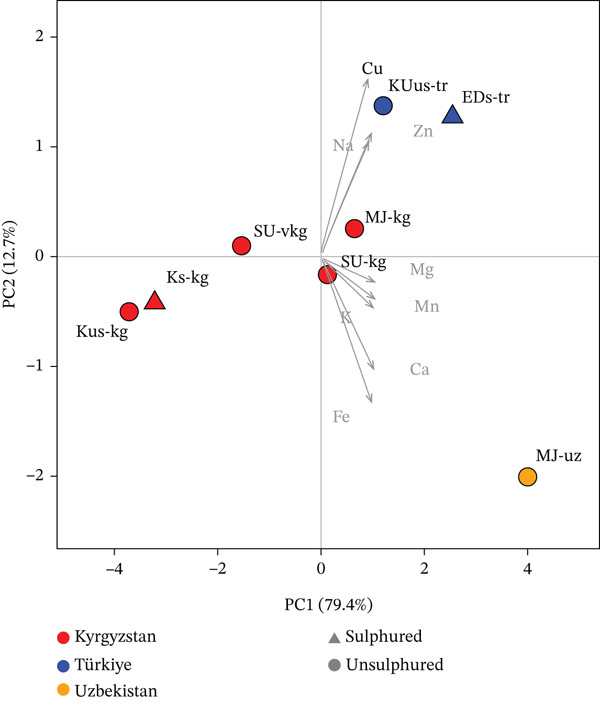
PCA—mineral composition (Ca, K, Mg, Na, Cu, Mn, Fe and Zn) of dried apricots (dry matter basis).

PCA of the eight measured elements (Ca, K, Mg, Na, Cu, Mn, Fe and Zn; sample means, *n* = 8, dry matter basis) yielded two components explaining 79.4% and 12.7% of the total variance (92.1% cumulative) (Figure 8). PC1 represented a general ‘mineral richness’ axis, on which all elements loaded positively with similar weight, clearly separating the mineral‐rich Uzbekistani sample (MJ‐uz) from the mineral‐poor Kurmaiy samples (Ks‐kg and Kus‐kg), irrespective of sulphuring status. PC2 further distinguished samples on the basis of relative Cu/Zn/Na versus Fe/Ca content, separating the Turkish samples from the remaining cultivars. As with the other variable blocks, sulphured and unsulphured samples of the same cultivar were not separated along either axis, confirming that mineral composition is primarily determined by geographical origin and cultivar rather than by sulphur dioxide treatment.

This integrative pattern is consistent with the block‐specific analyses above: mineral composition was most strongly associated with geographical origin, AA and sugar composition were more closely linked to cultivar, and antioxidant‐related compounds were most strongly influenced by sulphuring status.

## 4. Conclusion

Food composition data for sun‐dried, unsulphured organic and sulphured apricots were obtained using standard analytical methods. The results demonstrated significant variations in moisture content among the dried apricot samples, with apricots from Uzbekistan and Kyrgyzstan exhibiting substantially lower moisture content than those from Türkiye. Central Asian dried apricots were generally rich in carbohydrates, primarily sucrose. The total content of mono‐ and disaccharides in the analysed samples ranged from 31.6 to 57.9 g/100 g. The concentrations of sorbitol, sucrose and both micro‐ and macroelements varied according cultivars and geographical origin. Sun‐drying did not preserve ascorbic acid content or antioxidant capacity of the apricots. Nonetheless, dried apricots remain a valuable source of K and Mg, essential minerals associated with blood pressure regulation and cardiovascular health, which may contribute to a reduced risk of heart disease. Compressive knowledge of dried apricots composition offers important insights into their nutritional quality, potential health benefits and role in food security initiatives. The nutritional composition data generated in this study can be integrated into international food composition databases, including FoodEXplorer (EuroFIR), as dried apricots from Central Asia are increasingly imported into European markets.

## Author Contributions

**Jamila Smanalieva:** conceptualisation, methodology, investigation, data validation, writing – original draft preparation, writing – reviewing and editing. **Janyl Iskakova:** conceptualisation, methodology, investigation, validation, writing – review and editing. **Nurzat Shaikieva:** investigation, data validation, writing – review and editing. **Anke Foerster:** methodology, investigation, data validation, writing – review and editing. **Anne Hellwig:** methodology, investigation, data validation, writing – review and editing. **Uwe Schwarzenbolz:** methodology, data validation, writing – review and editing. **Thomas Henle:** conceptualisation, resources, writing – review and editing.

## Funding

This study was supported by the German Federal Ministry of Research, Technology and Space (Grant Number FKZ: 01LZ2201B). Open access funding was enabled and organized by Projekt DEAL.

## Conflicts of Interest

The authors declare no conflicts of interest.

## Data Availability

The data that support the findings of this study are available from the corresponding author upon reasonable request.
